# Multi-Omics Applications in Adult Acute Lymphoblastic Leukemia: From Biological Mechanisms to Precision Therapies

**DOI:** 10.3390/ijms27073335

**Published:** 2026-04-07

**Authors:** Claudia Simio, Matteo Molica, Laura De Fazio, Marco Rossi

**Affiliations:** Department of Hematology-Oncology, Azienda Universitaria Ospedaliera Renato Dulbecco, 88100 Catanzaro, Italyrossim@unicz.it (M.R.)

**Keywords:** adult acute lymphoblastic leukemia, multi-omics integration, genomic and transcriptomic profiling, epigenetic regulation

## Abstract

Adult acute lymphoblastic leukemia (ALL) is a highly heterogeneous hematologic malignancy where treatment response and relapse risk do not exclusively rely on the identification of genetic lesions but also on dynamic biological states sustained by specific transcriptional and epigenetic programs. Although the integrated application of multi-omics approaches has significantly expanded our knowledge of oncogenic dependencies, cellular plasticity, and mechanisms of therapeutic resistance, its systematic translation into the clinical practice of adult ALL is yet to become a reality. The aim of this review is to provide a critical and focused synthesis on how the integration of genomics, transcriptomics, and epigenetics enables the interpretation of disease biological behaviors and may guide personalized therapeutic strategies while simultaneously addressing the major limitations that hinder clinical implementation. Genomics allows for the identification of driver events and pharmacologically actionable vulnerabilities, whereas transcriptomics, including single-cell analyses, reveals functional states associated with clonal persistence, glucocorticoid resistance, and therapeutic adaptation, even in the absence of new mutations. In parallel, epigenetic signatures emerge as key elements in stabilizing oncogenic programs and resistant phenotypes, contributing to the biological plasticity of leukemic cells and representing potentially reversible therapeutic targets. Taken together, multi-omics signatures provide an integrated functional readout of adult ALL and support a dynamic precision-medicine model. However, adaptive therapeutic decisions aimed at relapse prevention require the full integration of these approaches through standardized strategies, longitudinal studies, and a sustainable implementation of molecular profiling and minimal residual disease monitoring.

## 1. Introduction

The treatment of acute lymphoblastic leukemia (ALL) represents a major clinical challenge in adults. Despite advances in chemotherapy protocols and the introduction of innovative immunotherapies, relapse and treatment resistance remain clinically relevant events, particularly in high-risk subtypes. These therapeutic failures largely reflect the biological complexity of the disease, which is characterized by marked heterogeneity and is mainly distinguished into B-ALL and T-ALL, with significant differences in clinical presentation, disease evolution, and therapeutic response [1].

In recent years, the application of multi-omics approaches—encompassing genomics, transcriptomics, and epigenetics—has revolutionized the understanding of the pathogenetic mechanisms underlying the disease and reflecting the biological plasticity of leukemic cells, defined as their ability to reversibly adapt transcriptional and phenotypic programs in response to environmental and therapeutic pressures.

In this review, we focus on the integration of multi-omics approaches—genomics, transcriptomics, and epigenetics—with particular attention to single-cell technologies, aiming to delineate not only the main molecular drivers of adult ALL but also their functional and clinical consequences. Our goal is to provide a dynamic and translational perspective that links cellular heterogeneity to precision medicine and suggests adaptive therapeutic strategies based on multi-omics data.

**Figure 1:**
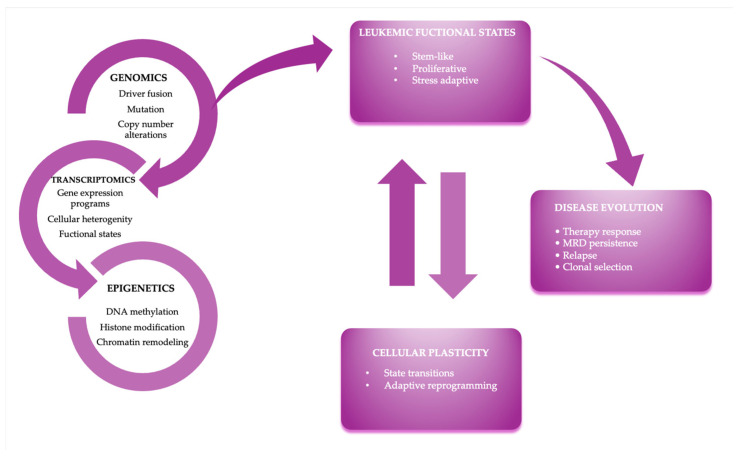
Integrated multi-omics framework linking leukemic functional states, cellular plasticity, and disease evolution in adult ALL. Genomic, transcriptomic and epigenetic alterations interact to shape functional leukemic states. Abbreviations: MRD, minimal residual disease.

Genomic studies have identified key subtypes defined by oncogenic fusions (e.g., BCR::ABL1 and Ph-like), recurrent mutations (TP53, NOTCH1, and IL7R), and copy-number alterations associated with prognosis and treatment response [2,3]. Transcriptomic analyses and single-cell RNA-seq have further highlighted intratumoral heterogeneity, identifying molecular signatures associated with drug resistance and potential therapeutic vulnerabilities. In parallel, epigenetic studies have shown how histone modifications, DNA methylation, and chromatin remodeling contribute to gene expression modulation of leukemic drivers [4,5]. Based on these considerations, Figure 1 provides an integrated conceptual framework, illustrating how genomic, transcriptomic, and epigenetic layers converge to define leukemic functional states, cellular plasticity, and disease evolution under therapeutic pressure in adult ALL.

Table 1 summarizes the main multi-omics layers and analytical approaches currently applied in adult ALL, highlighting their specific contribution to disease characterization and clinical interpretation.

## 2. Putative Clinical Workflow of Multi-Omics in Acute Lymphoblastic Leukemia

In clinical practice, the multi-omics approach in ALL is a longitudinal integrated process. At diagnosis, the integration of DNA- and RNA-based genomic and transcriptomic analyses focuses on the identification of oncogenic fusions, major molecular alterations and copy number alterations, in combination with conventional cytogenetics [6,7].

In a subsequent phase, initial genomic and transcriptomic information is used for risk stratification and to define the therapeutic strategy, guiding the selection of intensive regimens or the use of targeted therapies and immunotherapies in subgroups characterized by defined oncogenic dependencies [8].

During treatment, the monitoring of MRD by multiparametric flow cytometry, PCR, and high-sensitivity NGS technologies becomes a particularly relevant prognostic and decision-making tool when interpreted in light of the baseline molecular profile [9,10].

Indeed, integration between MRD and genomic data makes it possible to refine the interpretation of remission.

**Figure 2:**
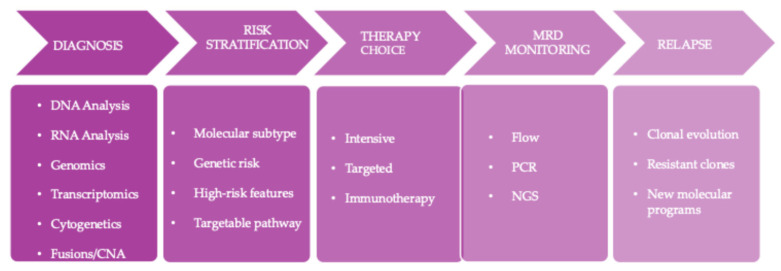
Multi-omics-guided clinical workflow in acute lymphoblastic leukemia. Integration of genomic, transcriptomic, and epigenetic analyses across disease stages supports diagnosis, risk stratification, treatment selection, and monitoring. Abbreviations: CNA, copy number alteration; PCR, polymerase chain reaction; NGS, next-generation sequencing.

In cases of persistent disease or relapse, molecular reassessment using multi-omics approaches enables the identification of clonal evolution, emergence of resistant subpopulations, and reactivation or acquisition of new transcriptional and epigenetic programs (Figure 2) [11].

## 3. Genomic Landscape and Clinical Implications

ALL is characterized by complex genetic heterogeneity, which translates into different clinical behaviors, therapeutic responses, and prognosis. Oncogenic fusions represent central events in pathogenesis and risk stratification [12,13]: in addition to the well-known *BCR::ABL1* fusion typical of Philadelphia-positive ALL (Ph+ ALL), numerous other relevant events have been identified, such as *ETV6::RUNX1* fusions, *KMT2A*-rearrangements, *DUX4*-rearranged, *MEF2D*-rearranged, and Ph-like forms, characterized by transcriptional activation profiles overlapping with Ph+ ALL and activating ABL or JAK-STAT pathways. These alterations define distinct molecular subtypes with specific clinical–therapeutic features: for example, Ph+ or Ph-like patients have an unfavorable prognosis without targeted treatment, whereas some fusions, such as *ETV6::RUNX1*, predominantly observed in pediatric ALL, are associated with a favorable response to conventional chemotherapy mainly in pediatric cohorts [14].

Alongside fusions, recurrent mutations contribute to disease pathogenesis and prognosis. TP53 mutations are associated with chemotherapy resistance and high relapse risk, reflecting impaired DNA-damage response, whereas alterations in *NOTCH1*, *IL7R*, *FBXW7*, and *PTEN*, particularly frequent in T-ALL, modulate proliferation, survival, and sensitivity to newer targeted agents through persistent activation of pro-survival and pro-proliferative pathways [15]. Copy number variations (CNVs), including deletions of genes such as *IKZF1*, *CDKN2A/B*, and *PAX5*, represent important prognostic markers and correlate with early relapse and pharmacologic resistance, even in patients in deep remission.

In addition to their descriptive identification, these genomic alterations exert specific functional effects on leukemic cell biology. *BCR::ABL1* and Ph-like fusions activate ABL and JAK-STAT signaling pathways, driving proliferation, survival, and resistance to apoptosis while sustaining transcriptional programs that promote oncogenic growth [7,14]. *KMT2A* rearrangements reorganize the epigenetic landscape, particularly through recruitment of DOT1L, leading to persistent activation of *HOXA* and *MEIS1* transcriptional networks, which block differentiation and maintain highly proliferative states [6,14]. *TP53* mutations impair DNA-damage response pathways, reducing apoptosis and enabling the survival of resistant subclones, with downstream effects on stress-response transcriptional programs [2,7]. In T-ALL, *NOTCH1* and *FBXW7* alterations activate *MYC* and *PI3K/AKT/mTOR* pathways, enhancing proliferation and maintaining quiescent or stem-like leukemic states, while IL7R mutations promote JAK-STAT signaling and expansion of pro-survival transcriptional programs. Finally, copy-number variations, such as deletions of IKZF1, CDKN2A/B, and PAX5, disrupt differentiation and cell-cycle control, reinforcing immature and therapy-resistant cellular states [16,17].

The combination of fusions, mutations, and CNVs has enabled the definition of distinct molecular subtypes of adult ALL with specific clinical–prognostic profiles [16,17].

For instance, adult patients with *BCR::ABL1*-positive ALL benefit from the early integration of tyrosine kinase inhibitors into chemotherapy regimens, resulting in improved remission rates and survival outcomes. Similarly, the identification of Ph-like ALL through genomic profiling has enabled the recognition of patients who may benefit from targeted inhibition of kinase-activating pathways, despite the absence of the canonical *BCR::ABL1* fusion [18].

Genomic characterization of this subtype has provided the biological rationale for the development of menin-KMT2A inhibitors, currently under clinical investigation. In this setting, molecular definition extends beyond prognostic stratification and enables exploration of subtype-specific targeted strategies.

Genomic identification of this entity may prompt more aggressive therapeutic approaches or early consideration of allogeneic stem cell transplantation in first complete remission, illustrating how genomic profiling directly informs high-impact clinical decisions.

In T-ALL, activating *NOTCH1* mutations or alterations in *FBXW7* define biologically distinct subgroups with prognostic and potentially therapeutic implications. Aberrant NOTCH signaling has provided the rationale for investigational use of γ-secretase inhibitors or strategies targeting downstream pathways, highlighting how genomic characterization can guide the development of targeted approaches even in the absence of established approved therapies.

This genomic classification guides risk stratification, identification of patients eligible for targeted therapies, and the selection of personalized clinical protocols, transforming the initial genomic profile into a clinically relevant decision-making tool [19,20,21].

Indeed, clonal dynamics limits the value of a static genomic evaluation at diagnosis and supports the use of longitudinal analyses and single-cell sequencing, which allow for the mapping of clonal evolution and identification of resistant subpopulations and provide fundamental information to optimize therapeutic strategies and anticipate treatment failures.

Sequencing studies show that relapse-driving clones may arise from early founder populations, highlighting the limits of static genomic analysis [22,23].

## 4. Transcriptomics and Molecular Profiling

Transcriptomic analysis links genomic subtypes with gene expression patterns and pathways influencing treatment response [24,25,26].

Indeed, patients with similar genomic risk profiles may experience markedly different clinical courses because of divergent transcriptional [27,28,29] and epigenetic programs that govern therapy adaptation and survival under treatment pressure [30,31]. In this setting, transcriptomic profiling helps to identify leukemic states associated with MRD persistence, early treatment failure, or poor response to standard chemotherapy, even in the absence of high-risk genomic lesions [32,33].

Major transcriptomic strategies include bulk RNA-seq, which has been extensively applied in adult ALL [34,35].

Bulk RNA-seq studies in ALL have revealed transcriptional signatures reflecting activation of critical biological pathways. For instance, Ph-like ALL—initially characterized in pediatric and AYA cohorts and subsequently confirmed in adult ALL—shows upregulation of JAK–STAT and ABL signaling pathways, consistent with the presence of fusions activating these pathways and with sensitivity to JAK or ABL inhibitors [36,37,38]. These expression profiles provide a strong biological rationale for the use of targeted therapies even in the absence of identifiable canonical genetic alterations, reinforcing the functional value of transcriptomic analysis [25,26,27].

Beyond molecular subtype definition, bulk RNA-seq has enabled the identification of transcriptional programs associated with drug resistance [27,28,29].

Furthermore, bulk transcriptomic analyses have identified stress-adaptive and inflammatory transcriptional states enriched in patients with persistent minimal residual disease or early relapse. These programs involve interferon signaling, metabolic rewiring, and survival pathways and are not consistently predictable from genomic alterations alone [31,32,33].

Similarly, in T-ALL, transcriptional profiles enriched for genes involved in *NOTCH1*, *PI3K/AKT/mTOR*, and *MYC* signaling are associated with a highly proliferative phenotype and increased resistance to conventional chemotherapy, highlighting the relevance of these pathways as potential therapeutic targets [34,35].

Single-cell RNA-seq analyses have demonstrated the coexistence of transcriptionally distinct cellular clusters within the same patient across pediatric, AYA, and adult ALL cohorts, revealing functional heterogeneity that is not captured by bulk approaches [36,37]. Among these, specific subpopulations exhibit activation of stress-adaptive and pro-survival transcriptional programs, including marked upregulation of *HSP90* and *FOXM1* [38,39].

Consequently, standard genomic profiling at diagnosis would not predict the emergence or expansion of these stress-adaptive subpopulations [40,41].

Single-cell transcriptomic studies have shown that specific leukemic cell states characterized by quiescent or stress-adaptive transcriptional programs are associated with increased disease persistence and inferior clinical outcomes, independently of the underlying genetic profile, supporting a direct prognostic impact of these functional configurations [42,43].

Collectively, these data suggest that persistence of specific transcriptional states, rather than acquisition of new genetic lesions, may contribute to treatment failure and relapse [44,45].

From a clinical perspective, these findings support the notion that MRD reflects the persistence of leukemic subpopulations defined by adaptive transcriptional states, which may be detectable at diagnosis or early during treatment [35].

These studies provide direct evidence that relapse in ALL can be primarily driven by transcriptional and epigenetic reprogramming, enabling leukemic cells to adapt to therapy without requiring additional genetic lesions [26]. As a result, static genomic profiling alone, while essential for initial disease classification, is insufficient to predict treatment resistance and relapse, highlighting the importance of integrating functional layers of information to capture leukemic cell behavior under therapeutic pressure [19,20].

## 5. Epigenetics: Mechanisms and Therapeutic Targets

Epigenetics plays a central role in the pathogenesis of adult ALL, regulating the expression of driver genes without altering the DNA sequence. Major alterations include DNA methylation, histone modifications, and chromatin remodeling, which maintain aberrant transcriptional programs promoting proliferation, leukemic survival, and treatment resistance [46].

### 5.1. DNA Methylation

Methylation of CpG promoters is one of the most studied epigenetic mechanisms in ALL. Promoters of tumor-suppressor genes such as *CDKN2A*/*B*, *TP53*, *IKZF1*, and *PAX5* can be hypermethylated, leading to silencing and loss of control over the cell cycle and apoptosis [47].

In B-ALL, this phenomenon influences resistance to glucocorticoids and chemotherapy, reducing sensitivity to conventional agents [36,47].

In T-ALL, aberrant promoter methylation of *BCL2L11* (BIM) and *PTEN* contributes to refractoriness to glucocorticoids and intensive chemotherapy, often in cooperation with activating *NOTCH1* mutations, strengthening pro-survival signals and limiting apoptosis induction [48].

### 5.2. Histone Modifications and Chromatin Remodeling

Histone modifications regulate chromatin structure and gene accessibility, influencing transcriptional activation or repression.

Key enzymes:

- HDACs and HATs regulate expression of tumor-suppressor genes and influence response to therapy [40,49].
- *DOT1L* (*H3K79* methyltransferase) and *EZH2* (*H3K27* trimethyltransferase) maintain oncogenic transcriptional programs, particularly in subtypes characterized by specific epigenetic dependencies [50].

In *KMT2A*-rearranged T-ALL subtypes, hyperactivity of *HDACs* or *DOT1L* preserves an aberrant chromatin state, promoting proliferation, survival, and pharmacologic resistance.

In B-ALL, aberrant histone modifications may silence genes controlling differentiation and apoptosis, reducing response to therapy [51].

### 5.3. Role of Epigenetic Alterations in Drug Resistance

Epigenetic alterations modulate therapeutic response in both B-ALL and T-ALL.

#### 5.3.1. *B-ALL*

- Glucocorticoid resistance: Hypermethylation of BIM (*BCL2L11*) and *CDKN2A/B* promoters, together with repression of *NR3C1*, reduces prednisolone- or dexamethasone-induced apoptosis. This epigenetic profile favors leukemic-cell survival during early treatment phases [41].
- Chemotherapy resistance: Aberrant *H3K27* trimethylation mediated by *EZH2* and *DOT1L* hyperactivity maintains expression of oncogenes such as *HOXA9*, *MEIS1*, and *MYC*, reducing sensitivity to alkylating and nucleoside agents. *KMT2A*-rearranged clones show reduced apoptosis in response to daunorubicin or cytarabine. These mechanisms contribute to the persistence of refractory subclones [52].
- In Ph+ and Ph-like ALL, epigenetic alteration reduces dependence on ABL/JAK signaling, promoting TKI resistance through upregulation of compensatory pathways such as *PI3K*/*AKT*/*mTOR* or *BCL2*. This phenomenon explains the loss of efficacy of targeted therapies in the absence of new resistance mutations [53].

#### 5.3.2. *T-ALL*

- Resistance to glucocorticoids and intensive chemotherapy: Dysregulation of *EZH2*, *PRC2*, and *HDAC* supports pro-survival programs, while quiescent “stem-like” subclones activate stress response genes (*HSP90* and *FOXM1*) and anti-apoptotic factors (*BCL2*, *MCL1*) that persist during therapy. These cell populations represent a potential reservoir for relapse [54].
- Epigenetic modifications interact with mutations or fusions (e.g., *NOTCH1*, *IL7R*, and *BCR: ABL1*), stabilizing oncogenic programs and contributing to refractoriness. This crosstalk strengthens the functional dependence of leukemic cells on specific biological programs [55].

### 5.4. Therapeutic Targets and Clinical Approaches

Epigenetic alterations represent potentially reversible therapeutic targets and have, therefore, attracted interest for precision medicine strategies in ALL.

A growing number of epigenetic modulators are available, spanning agents with a strong preclinical rationale, compounds supported by early-phase clinical data in ALL, and drugs approved in other hematologic malignancies but not yet established in ALL [56] (Table 2).

Distinct classes of epigenetic inhibitors target different layers of chromatin regulation, including DNA methylation, histone acetylation and methylation, and chromatin-associated cofactors. While these approaches have demonstrated the ability to reverse transcriptional repression programs, restore tumor suppressor gene expression, and sensitize leukemic cells to cytotoxic or targeted agents, their clinical translation faces several challenges. These include dose-limiting toxicities, a lack of robust predictive biomarkers for patient selection, and uncertainty regarding optimal combinatorial strategies [57].

#### 5.4.1. DNA Methylation Inhibitors (DNMTi)

Hypomethylating agents such as 5-azacitidine and decitabine inhibit DNA methyltransferases through incorporation into DNA, leading to passive demethylation of silenced tumor suppressor genes. Preclinical studies suggest that DNMTi may reverse aberrant methylation of genes involved in apoptosis and glucocorticoid response, thereby restoring chemosensitivity [56].

Early studies have mainly explored DNMTi in combination regimens as epigenetic priming strategies rather than standalone therapies [56].

#### 5.4.2. Histone Deacetylase Inhibitors (HDACi)

Histone deacetylase inhibitors, including vorinostat, romidepsin, panobinostat, and entinostat, promote chromatin relaxation and re-expression of transcriptionally repressed genes. In adult ALL, however, clinical data remain limited and largely restricted to early-phase or exploratory studies. HDACi are generally considered adjunctive agents, used in combination with chemotherapy or targeted therapies to enhance efficacy, rather than standalone treatments [57]. Notably, concerns regarding hematologic toxicity and off-target effects have limited their broader adoption.

#### 5.4.3. Inhibitors of Specific Histone Methyltransferases (HMTi)

Targeting histone methylation represents a more subtype-specific epigenetic strategy. Pinometostat (EPZ-5676), a DOT1L inhibitor, has shown biological and early clinical activity in leukemias harboring *KMT2A* rearrangements by suppressing *HOXA* and *MEIS1*-driven transcriptional programs. While this represents one of the most mechanistically compelling epigenetic approaches in ALL, clinical responses have been modest and transient, highlighting the need for combination strategies [58].

*EZH2* inhibitors, such as tazemetostat, target aberrant *H3K27* trimethylation mediated by PRC2 complexes [59].

#### 5.4.4. Arginine Methylation Inhibitors (PRMTi)

Protein arginine methyltransferase inhibitors, particularly *PRMT5* inhibitors such as GSK3326595, are under clinical investigation in other hematologic malignancies.

In ALL, evidence remains largely preclinical and suggests that inhibition of arginine methylation may disrupt transcriptional and stress-adaptive programs [59].

#### 5.4.5. Targeting Epigenetic Cofactors and Combinatorial Approaches

Inhibition of chromatin-associated cofactors represents an emerging therapeutic paradigm. Menin inhibitors, such as revumenib, indirectly disrupt KMT2A-driven transcriptional complexes and have shown promising activity in *KMT2A*-rearranged acute leukemias, including ALL [61]. Although not classical epigenetic inhibitors, these agents exemplify how targeting epigenetic dependencies can yield clinically meaningful responses [60].

Given the adaptive nature of epigenetic plasticity, combinatorial approaches are increasingly viewed as essential. Preclinical studies indicate synergistic effects when *DNMTi* are combined with *HDACi* or integrated with conventional chemotherapy, tyrosine kinase inhibitors, or BH3 mimetics. The identification of predictive biomarkers remains a major unmet need and a critical barrier to the routine incorporation of epigenetic therapies into precision treatment algorithms for adult ALL.

**Figure 3:**
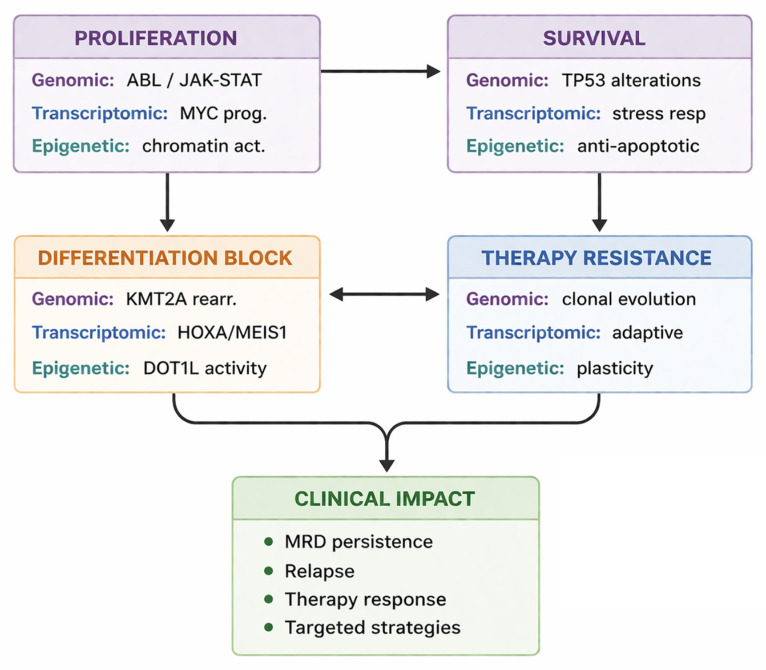
Integrated molecular programs driving leukemic plasticity, therapy resistance, and clinical outcome in adult ALL. Abbreviations: MRD, minimal residual disease.

The integrated functional impact of genomic, transcriptomic, and epigenetic alterations on leukemic cell states and clinical outcome is schematically summarized in Figure 3.

## 6. Multi-Omics Integration in Adult ALL

Genomic alterations define founding events of leukemogenesis, whose clinical impact emerges through transcriptional programs and epigenetic regulation. Multi-omics enables an integrated interpretation of leukemic cell behavior under therapeutic pressure.

Recently, the use of single-cell technologies, such as scRNA-seq and scATAC-seq, has enabled the mapping of intra-leukemic heterogeneity and the identification of rare therapy-resistant subpopulations that are not detectable by bulk analyses [62,63]. These data can be integrated using platforms such as Seurat, MOFA+, or LIGER to correlate transcriptional states with genomic and epigenetic profiles [62]. The single-cell approach enables the tracing of functional trajectories, the identification of adaptive clones, and the prediction of potential resistance mechanisms. Integrating these data with bulk and longitudinal datasets provides a dynamic view of leukemic behavior under therapeutic pressure, supporting adaptive treatment strategies and the selection of targeted combination therapies. While these analyses remain primarily in the research setting, they represent a crucial step toward the implementation of a dynamic multi-omics–based precision medicine model.

In Ph+ and Ph-like ALL, the presence of *BCR::ABL1* fusions or alterations activating ABL or JAK-STAT pathways is associated with transcriptional profiles characterized by genes involved in proliferation, survival, and cell-cycle regulation. Integrative studies have shown that these transcriptional programs are supported by epigenetic remodeling processes that contribute to stabilization of the oncogenic state, progressively limiting the effectiveness of tyrosine kinase inhibitors and favoring the emergence of adaptive resistance mechanisms even in the absence of new mutations [14,26].

Clinical studies have demonstrated the efficacy of combining tyrosine kinase inhibitors with immunotherapy, such as dasatinib–blinatumomab- or ponatinib-based regimens, significantly improving outcomes in adult Ph+ ALL [64,65].

A similar model is observed in *KMT2A*-rearranged ALL, in which the primary genomic lesion is associated with profound reorganization of the epigenetic landscape and persistent activation of *HOXA* and *MEIS1* transcriptional programs. In this subgroup, the interplay between genetic, epigenetic, and transcriptional programs explains the biological aggressiveness of the disease and its limited sensitivity to conventional chemotherapy while simultaneously providing the rationale for therapeutic strategies targeting specific epigenetic vulnerabilities [7,38,50].

Epigenetic therapies targeting specific vulnerabilities, such as DOT1L inhibition, have shown clinical activity in selected ALL subgroups [58].

In adult T-ALL, aberrant activation of *NOTCH1*, *IL7R*, or the JAK-STAT and *PI3K/AKT/mTOR* pathways defines relevant oncogenic dependencies; however, treatment response is often influenced by heterogeneous functional states of leukemic cells [16,26,39]. Transcriptomic analyses, particularly at the single-cell level, have revealed subpopulations with quiescent or stress-response programs, frequently associated with epigenetic repression of apoptotic pathways. These cellular states may persist during therapy and contribute to MRD and relapse.

Recent multimodal single-cell studies have highlighted the contribution of both genetic and non-genetic mechanisms to early drug resistance and disease persistence [62].

**Figure 4:**
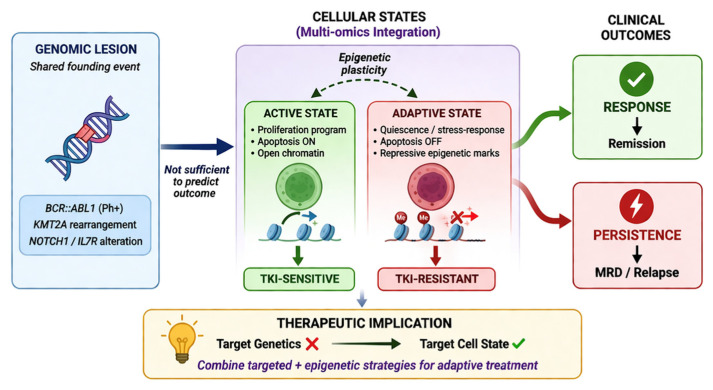
Dynamic interplay of multi-omics layers driving leukemic evolution in adult ALL. A multi-omics perspective reveals that identical genomic lesions in adult ALL can generate distinct cellular states through transcriptional and epigenetic regulation. Abbreviations: TKI, tyrosine kinase inhibitor; MRD, minimal residual disease; Ph+, Philadelphia chromosome-positive.

Multi-omics enables dynamic disease management and adaptive therapeutic strategies based on leukemic evolution (Figure 4).

## 7. Multi-Omics Integration and Clinical Applications

In adult ALL subtypes characterized by defined oncogenic dependencies, integration of genomic, transcriptomic, and epigenetic data has already led to substantial changes in therapeutic strategies and response monitoring [63].

In this context, multi-omics acts as an interpretative tool linking molecular alterations to personalized therapeutic decisions. This approach has proven particularly effective in B-ALL and is progressively emerging in T-ALL (Table 3).

In Ph+ B-ALL, genomic identification of the *BCR::ABL1* fusion has led to the development of therapeutic strategies based on ABL-class inhibitors, validated in prospective clinical studies. The Italian D-ALBA trial [64] demonstrated that the combination of dasatinib followed by blinatumomab, without conventional chemotherapy, induces high rates of deep molecular remission and prolonged event-free survival. Similarly, studies conducted at the MD Anderson Cancer Center showed that the combination of ponatinib + blinatumomab enables MRD-negativity rates above 80% in adults with Ph+ ALL, reducing the need for allogeneic transplantation in first remission [65].

A second paradigm of multi-omics-guided precision medicine is represented by Ph-like B-ALL. Integrated genomic analyses subsequently showed that these cases frequently harbor *CRLF2* rearrangements, *JAK2* mutations, or *ABL*-class fusions.

Combined genomic and transcriptomic profiling enables identification of actionable signaling dependencies.

Based on such evidence, the North American COG AALL1521 trial evaluated the addition of ruxolitinib to conventional chemotherapy in patients with JAK-STAT pathway activation, while other studies explored the use of dasatinib or ponatinib in subgroups with ABL-class fusions [66,67].

These studies provide a biological rationale for targeted therapy in this high-risk subtype.

In relapsed/refractory B-ALL, the ZUMA-3 trial showed that brexucabtagene autoleucel induces complete remission in about 70–80% of patients.

Integrated translational analyses have shown that transcriptomic and epigenetic features of leukemic cells and of the immune microenvironment influence depth and durability of response, as well as escape mechanisms, supporting integration of multi-omics in selection and timing of immunotherapy. Multi-omics may help identify mechanisms of immune escape and guide combinatorial strategies.

The prognostic and decision-making value of multi-omics integration also clearly emerges in MRD monitoring. Studies conducted by Logan and colleagues [67,68] showed that MRD assessed by NGS (ClonoSEQ), integrated with the initial mutational profile, provides superior prediction of relapse risk compared with flow cytometry or conventional PCR. Combined analysis of MRD and high-risk genomic alterations improves relapse risk prediction. In adult T-ALL, clinical application of multi-omics is less advanced but supported by solid molecular studies. Genomic and transcriptomic analyses have identified subtypes characterized through aberrant activation of *NOTCH1*, *IL7R*/*JAK-STAT*, or *PI3K*/*AKT*/*mTOR*, often associated with epigenetic programs of glucocorticoid resistance. Phase I/II clinical studies with gamma-secretase inhibitors (Clinical trial NCT00878189) have provided a proof-of-concept for *NOTCH1* targeting, although toxicity has limited clinical applicability [54,55]. In parallel, international translational studies are evaluating emerging immunotherapies and CAR-T-cells directed against T-cell-specific antigens (CD7, CD5), with preliminary results reported in phase I trials (clinical trial NCT04594135). Integration of multi-omics data may help identify patients eligible for targeted therapies.

This model enables translation of molecular complexity into concrete clinical decisions, making precision medicine an adaptive rather than a static process. Linking molecular alterations to targeted therapies and clinical trials represents a major advance of the omics era in ALL.

**Table 3:** Clinical paradigms of multi-omics-guided precision medicine in adult ALL. Abbreviations: Ph+, Philadelphia chromosome-positive; Ph-like, Philadelphia-like; ALL, acute lymphoblastic leukemia; MRD, minimal residual disease; NGS, next-generation sequencing; TKI, tyrosine kinase inhibitor; CAR-T, chimeric antigen receptor T-cells.

Clinical Context	Integrated Omics Features	Therapeutic Implication	Clinical Relevance
Ph+ B-ALL	*BCR::ABL1* fusion; MRD by NGS	TKI + blinatumomab; chemo-free regimens [64,65]	Deep molecular remission; reduced transplant need [64,65]
Ph-like B-ALL	Ph-like transcriptomic signature; JAK/ABL alterations	JAKi or ABL-class inhibitors with chemotherapy [68]	Targeted therapy in high-risk subtype [68]
Relapsed/ Refractory B-ALL	Transcriptomic: epigenetic immune-escape programs	CAR-T or bispecific antibodies [67]	Improved response selection and durability [67]
MRD-negative but high-risk ALL	MRD negativity; Adverse genomic profile	Treatment intensification or adaptive strategies [68]	Improved relapse prediction [68]
Adult T-ALL	NOTCH1/JAK-STAT activation; epigenetic resistance programs	Experimental targeted or immunotherapy approaches [54,55]	Identification of candidates for trials [54,55]

## 8. Clinical Challenges, Future Perspectives and Conclusions

The application of multi-omics approaches has provided an integrated and systemic view of adult ALL, clarifying how the disease emerges from the dynamic interaction between genomic alterations, aberrant transcriptional programs, and epigenetic deregulation.

Despite these advances, the standard of care in adult ALL remains predominantly anchored to a static precision medicine model, in which molecular characterization is largely confined to diagnosis.

A major limitation of current multi-omics studies in ALL is their predominant reliance on pediatric or AYA cohorts, which constrains biological interpretation in adult disease. These differences limit the direct transferability of pediatric-derived models and underscore the urgency of prospective studies specifically dedicated to the adult population.

The evidence discussed in this review supports a dynamic precision medicine model, in which genomics, transcriptomics, and epigenetics act as interconnected layers shaping disease behavior over time. Genomic alterations define founding events and oncogenic dependencies; transcriptional programs capture functional and adaptive cellular states; and epigenetic mechanisms stabilize gene-expression patterns associated with disease persistence and therapeutic resistance. These layers cooperate in determining leukemic cell responses to therapy and their evolution during treatment. Their integrated interpretation provides the biological bases to explain how comparable clinical responses may subsequently evolve into divergent outcomes.

Nevertheless, these applications remain largely episodic and do not yet constitute a fully implemented dynamic clinical strategy.

The translation of a dynamic precision medicine model into routine clinical practice is hindered by several challenges. Methodological heterogeneity and a lack of standardization across platforms and bioinformatic pipelines limit reproducibility and data integration, directly affecting the feasibility of omics-guided risk stratification and therapeutic decision-making. In addition, the coexistence of multiple molecular alterations within the same patient complicates clinical interpretation, as not all detected events have biological or therapeutic relevance. Overcoming these limitations requires harmonization of analytical workflows, the development of clinically oriented multi-omics panels, and the definition of internationally shared molecular subtypes.

Accordingly, the most urgent step for the field is the prospective clinical validation of integrated longitudinal molecular monitoring strategies in adult ALL. Such strategies should combine NGS-based MRD assessment with transcriptomic and epigenetic profiling at defined clinical time points to identify early signals of adaptive resistance and guide timely treatment modulation. This transition from static, diagnosis-centered decision-making to adaptive treatment informed by biological evolution represents the critical bridge between multi-omics discovery and clinical impact.

Sustainable implementation will require centralized molecular platforms, simplified assays focused on clinically actionable biology, and the expansion of adaptive clinical trials embedded within collaborative networks.

**Figure 5:**
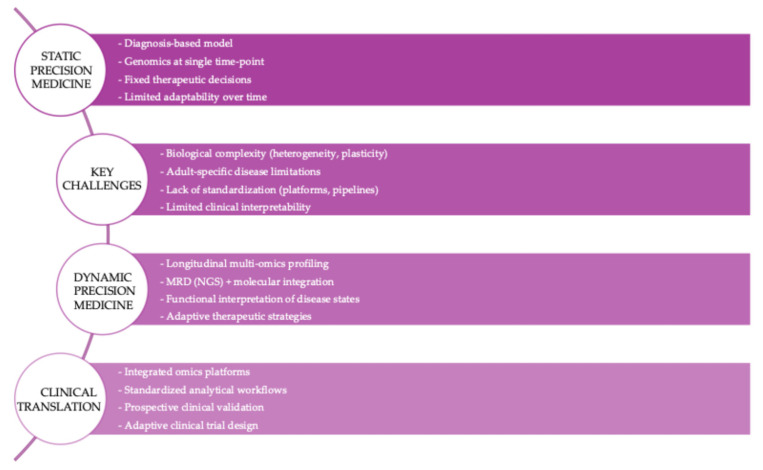
From static to dynamic precision medicine in adult ALL. Traditional approaches based on baseline genomic profiling are being replaced by dynamic models integrating longitudinal multi-omics data. Abbreviations: MRD, minimal residual disease; NGS, next-generation sequencing.

Multi-omics approaches are reshaping the biological understanding and clinical management of adult ALL, supporting a transition from static risk stratification to dynamic precision medicine strategies (Figure 5).

The effective implementation of this model represents a fundamental step toward more effective, personalized, and durable therapeutic strategies for patients with adult ALL.

## Figures and Tables

**Table 1:** Genomic, transcriptomic, and epigenetic features and clinical implications in B-ALL and T-ALL. Abbreviations: B-ALL, B-cell acute lymphoblastic leukemia; T-ALL, T-cell acute lymphoblastic leukemia.

Omics Layer	B-ALL—Functional Insight	T-ALL—Functional Insight	Clinical Relevance
Genomics	Subtype-defining driver lesions establish baseline risk (e.g., *BCR::ABL1*, *IKZF1*) [2]	Lineage specific oncogenic programs drive proliferation (e.g., *NOTCH1*) [3]	Risk stratification and targeted therapy selection [2,3,5]
Transcriptomics/single-cell	Coexistence of distinct functional states within the same leukemia (e.g., stem-like vs cycling programs) [2]	Dynamic transcriptional programs under treatment pressure [3]	Detection of resistant or persistent leukemic populations [2,3,5]
Epigenetics	Epigenetic programs support stability of leukemic cell states (e.g., DNA methylation patterns) [4]	Chromatin remodeling enables phenotypic plasticity (e.g., histone modifications) [4]	Potential reversibility of drug resistance [4,5]

**Table 2:** Epigenetic mechanisms and therapeutic targets in adult acute lymphoblastic leukemia. Abbreviations: DNMTs, DNA methyltransferases; GC, glucocorticoids; HDACs, histone deacetylases; DOT1L, disruptor of telomeric silencing 1-like; EZH2, enhancer of zeste homolog 2; PRC2, polycomb repressive complex 2; PRMT5, protein arginine methyltransferase 5; KMT2A-r, KMT2A-rearranged.

Epigenetic Mechanism	Key Targets/Enzymes	Biological Effect	Therapeutic Implication
DNA methylation	*DNMTs; BIM, CDKN2A/B; NR3C1*	Apoptosis silencing; GC resistance	DNMT inhibitors; chemosensitization [47,48]
Histone acetylation	*HDACs*	Repression of tumor suppressors	HDAC inhibitors (combinatorial use) [49,57]
H3K79 methylation	*DOT1L*	Maintenance of HOXA/MEIS1 programs	DOT1L inhibitors (*KMT2A*-r ALL) [50,58]
H3K27 trimethylation	*EZH2; PRC2*	Stabilization of oncogenic states	EZH2 inhibitors [51,59]
Chromatin cofactors	*Menin*	KMT2A-driven transcription	Menin inhibitors [60]
Epigenetic plasticity	*PRMT5; HDAC; EZH2*	Adaptive resistance states	Rational combinations [54,55,56]

## Data Availability

No new data were created or analyzed in this study. Data sharing is not applicable to this article.
